# Interpreting p53 IHC–TP53 NGS discordance in endometrial cancer: a mechanism-based diagnostic framework and clinical implications

**DOI:** 10.3389/fonc.2026.1862855

**Published:** 2026-07-22

**Authors:** Wiktor Szatkowski, Agnieszka Harazin-Lechowska, Tomasz Kluz, Aleksandra Dudek, Janusz Ryś, Paweł Blecharz, Małgorzata Nowak-Jastrząb, Andrzej Jasiewicz, Ewa Kaznowska, Ewa Samborska, Agnieszka Adamczyk

**Affiliations:** 1 Department of Gynaecologic Oncology, Maria Skłodowska-Curie National Research Institute of Oncology, Kraków, Poland; 2 Department of Pathology, Maria Skłodowska-Curie National Research Institute of Oncology, Kraków, Poland; 3 Department of Gynecology and Obstetrics, Institute of Medical Sciences, Medical College of Rzeszów University, Rzeszów, Poland; 4 Laboratory of Clinical Genetics, Molecular Biology and Translational Research, Faculty of Medicine, Rzeszów, Poland; 5 Department of Pathology, Faculty of Medicine, University of Rzeszów, Rzeszów, Poland

**Keywords:** discordance, endometrial cancer, immunohistochemistry, molecular classification, next-generation sequencing, p53, ProMisE, *TP53*

## Abstract

**Objective:**

To analyse discordances between p53 immunohistochemistry (IHC) and *TP53* next-generation sequencing (NGS) in endometrial cancer, with a focus on underlying mechanisms and their clinical implications. Despite increasing recognition of IHC–NGS discordance, a structured approach to its interpretation in routine practice remains lacking.

**Methods:**

A retrospective analysis was performed in 614 patients with endometrial cancer treated at two oncological centres between 2022 and 2025. All patients underwent both p53 IHC and *TP53* NGS. Molecular classification followed the ProMisE hierarchy (POLEmut, MMRd/MSI-H, p53abn, NSMP). The subgroup of 110 patients with p53 abnormalities identified by p53 IHC and/or *TP53* NGS (defined as an abnormal p53 IHC pattern, a *TP53* mutation detected by NGS, or both) was analysed to identify and mechanistically classify IHC–NGS discordant cases, as discordance carries the most direct therapeutic implications within this subtype. Associations between molecular subtype and disease stage were evaluated using logistic regression.

**Results:**

Among 110 patients with p53 abnormalities identified by p53 IHC and/or *TP53* NGS, 23 IHC–NGS discordances were identified (20.9%). The most frequent source of discordance was difficulty in interpreting IHC expression thresholds (14/23; 60.9%), particularly around the 1% and 80% cut-offs. Other mechanisms included intratumoral heterogeneity with subclonal p53 expression (n=6), low-VAF *TP53* mutations (n=2), and *TP53*-independent p53 stabilisation (n=1). Discordant cases were systematically organised into seven mechanistic groups forming a structured interpretative framework. In the full cohort, p53abn was the only subtype significantly associated with advanced-stage disease (OR 1.79; 95% CI 1.06–3.03; p=0.031).

**Conclusions:**

IHC–NGS discordance in p53 assessment represents a multimechanistic phenomenon observed in over 20% of p53-abnormal cases. The proposed mechanism-based interpretative framework may support consistent evaluation of borderline cases and improve diagnostic decision-making in clinical practice. In clinically significant scenarios, particularly near diagnostic thresholds, pathological re-evaluation and supplementary NGS testing should be considered prior to treatment planning.

## Introduction

The introduction of molecular classification of endometrial cancer and the updated FIGO 2023 staging system has fundamentally changed the approach to this disease, shifting the focus from anatomical assessment to tumour biology (1, 2, 34). The central element of this new paradigm is p53 protein status — a key regulator of genomic stability. Abnormal p53 status, reflected by the p53abn subtype, is associated with the worst prognosis amongst all four molecular subtypes and directly influences adjuvant treatment decisions (1, 2, 23). At the same time, this marker generates the most diagnostic and interpretive problems in daily practice, which may lead to both undertreatment and excessive intensification of therapy.

In clinical practice, the primary tool for p53 status assessment remains immunohistochemistry (IHC), used as a surrogate marker for *TP53* mutation. Although IHC concordance with next-generation sequencing (NGS) is reported as approximately 92–95%, a significant subset of cases remains diagnostically ambiguous (4, 5). Discordances may arise from subjectivity in staining pattern interpretation, limited IHC specificity for certain mutation types, intratumoral heterogeneity, and pre-analytical factors. A particular challenge is the interpretation of p53 expression threshold values — the lower 1% boundary distinguishing null phenotype from wild-type pattern, and the upper 80% boundary above which overexpression is recognised.

An additional layer of complexity arises from the co-occurrence of *TP53* abnormalities with other molecular aberrations. In such multiple-classifier tumours, strict adherence to the ProMisE hierarchy is critical (POLEmut > MMRd > p53abn), as secondary *TP53* mutations do not determine aggressive tumour biology and their misinterpretation may lead to unjustified treatment intensification (3, 12, 30, 31). The clinical consequences of misclassification are substantial: patients with true p53abn require platinum- and taxane-based chemotherapy, as demonstrated in PORTEC-3 and confirmed in its 10-year follow-up analysis, whilst external beam radiotherapy alone does not prevent distant recurrences that dominate in this subtype (7, 8, 26). Misclassification in either direction therefore directly affects patient outcomes.

The aim of this study was to analyse discordances between p53 IHC and *TP53* NGS in a cohort of endometrial cancer patients, focusing on the underlying mechanisms and their clinical implications.

## Materials and methods

### Study design and cohort

This study was conducted as a retrospective analysis of clinical and pathomorphological data from two oncological centres. Patients with histopathologically confirmed endometrial cancer operated between April 2022 and December 2025 were included. The total cohort comprised 614 patients. Collected data included age at surgery, histological type, tumour grade, lymphovascular space invasion (LVSI), depth of myometrial invasion, and disease stage according to FIGO 2023.

All 614 patients underwent both p53 immunohistochemistry (IHC) and *TP53* next-generation sequencing (NGS). A subgroup of 110 patients with p53 abnormalities identified by p53 IHC and/or *TP53* NGS was identified for discordance analysis; this status was defined as an abnormal p53 IHC pattern, a pathogenic or likely pathogenic *TP53* mutation detected by NGS, or both. Discordance analysis was restricted to this subgroup because IHC–NGS discordance carries the most direct therapeutic implications within the p53abn subtype. Within this subgroup, 23 cases (20.9%) with IHC–NGS discordance were identified and subjected to detailed mechanistic analysis.

The study was approved by the Bioethics Committee of the National Research Institute of Oncology in Warsaw (“Komisja Bioetyczna w Narodowym Instytucie Onkologii w Warszawie”) (No. 6/2025, 9 January 2025) and was conducted in accordance with the Declaration of Helsinki. Individual patient consent was waived due to the retrospective nature of the analysis.

### Molecular classification

Molecular classification followed the ProMisE hierarchy, distinguishing four subtypes: POLE-mutated (POLEmut), mismatch repair-deficient (MMRd/MSI-H), p53-abnormal (p53abn), and no specific molecular profile (NSMP). p53abn status was assigned when an abnormal p53 IHC pattern, a pathogenic or likely pathogenic *TP53* mutation by NGS, or both were identified. For discordant cases, p53 IHC patterns were used primarily to support mechanistic categorisation of discordance. Final molecular subtype assignment followed the integrated ProMisE framework, taking into account POLE status, MMR status, *TP53* sequencing results, and the overall biological interpretation of discordant findings.

### p53 immunohistochemistry

Immunohistochemical staining was performed on FFPE sections mounted on SuperFrost™ slides. Given the two-centre design, different IHC protocols were applied at each centre in accordance with locally validated procedures. At Centre 1, staining was performed on 3 μm sections using a monoclonal anti-p53 antibody (clone BP53-11, Ventana) in ready-to-use format on a Roche BenchMark ULTRA platform. At Centre 2, staining was performed on 4 μm sections using a monoclonal antibody (clone DO-7, Dako/Agilent, GA616/0) in ready-to-use format on a Dako Omnis platform. Both antibodies were applied in ready-to-use format and were not diluted.

p53 expression was classified as normal (wild type) or abnormal (p53abn) according to current criteria. The normal pattern was defined as nuclear staining of variable intensity in 1–80% of tumour cells. For the overexpression pattern, tumour cells with strong, diffuse nuclear staining in >80% of cells were distinguished from equivocal cases in which the estimated percentage of stained cells was in the range of 75–85% and the threshold could not be reliably determined; the latter were flagged for secondary pathological review by a pathologist with dedicated expertise in gynaecological pathology. Abnormal patterns included: overexpression (>80% of cells with strong nuclear staining), null phenotype (complete absence of nuclear staining with preserved internal control), cytoplasmic staining, and weak diffuse nuclear staining (blush pattern). The presence of two or more distinct patterns, each comprising ≥5% of tumour cells, was interpreted as evidence of molecular tumour heterogeneity.

### Next-generation sequencing

Genomic DNA was extracted from FFPE material following pathologist evaluation of slides to select blocks with adequate tumour cell content. Isolation was performed using the Maxwell^®^ RSC DNA FFPE Kit (Promega) on the Maxwell^®^ RSC platform. Sequencing was performed on the IonTorrent platform (Thermo Fisher Scientific) using a custom amplicon panel covering genes *BRCA1*, *BRCA2*, *MLH1*, *MSH2*, *MSH6*, *PIK3CA*, *PMS2*, *POLD1*, *POLE*, *TP53*, *CTNNB1*, and *KRAS* hotspots. Detected variants were classified per ACMG/AMP guidelines. Only pathogenic and likely pathogenic variants were included in clinical analyses. Mutation significance was assessed using publicly available tools and databases (wANNOVAR, VarSome, ClinVar).

### Definition and classification of discordances

Discordance was defined as disagreement in p53 status between IHC and *TP53* NGS in cases where both assessments were performed on material from the same tumour. Discordant cases were classified into seven mechanistic groups based on: the p53 IHC expression pattern, the type of *TP53* mutation in NGS, and the variant allele frequency (VAF).

### Statistical analysis

Categorical variables were presented as absolute numbers and percentages. Differences in the distribution of categorical variables between groups were assessed using the chi-squared test or Fisher’s exact test, as appropriate. The association between molecular subtype and disease stage was evaluated using univariable binary logistic regression, with advanced stage (FIGO III–IV) as the outcome and early stage (FIGO I–II) as the reference category. Results were expressed as odds ratios (OR) with 95% confidence intervals (CI). Statistical analyses were conducted using IBM SPSS Statistics and/or R. A two-sided p-value <0.05 was considered statistically significant.

## Results

The study included 614 patients operated on for endometrial cancer at two oncological centres. The mean age at surgery was 65.2 ± 9.9 years, with 33.4% of patients aged over 70 years. Endometrioid histology was confirmed in 554 cases (90.2%) and non-endometrioid in 57 (9.3%). Tumour grade was G1–2 in 508 cases (82.7%) and G3 in 86 (14.0%). Substantial LVSI was present in 132 patients (21.5%), and deep myometrial invasion (≥50%) in 248 (40.4%). Most patients presented with early-stage disease (FIGO I–II, 84.4%), whilst advanced stages (III–IV) were observed in 15.6%. Detailed clinicopathological characteristics are provided in **Table 1**.

**Table 1:** Clinicopathological characteristics of the study cohort (N = 614).

Parameter	Category	N (%) or mean ± SD
Age at surgery	Mean ± SD	65.2 ± 9.9 years
	< 60 years	181 (29.5%)
	60–70 years	225 (36.6%)
	> 70 years	205 (33.4%)
	Unknown	3 (0.5%)
Histology	Endometrioid	554 (90.2%)
	Non-endometrioid	57 (9.3%)
	Unknown	3 (0.5%)
Grade	G1–2	508 (82.7%)
	G3	86 (14.0%)
	Unknown	20 (3.3%)
LVSI	None/focal	476 (77.5%)
	Substantial	132 (21.5%)
	Unknown	6 (1.0%)
Myometrial invasion	< 1/2	359 (58.5%)
	≥ 1/2	248 (40.4%)
	Unknown	7 (1.1%)
FIGO stage	Early (I–II)	518 (84.4%)
	Advanced (III–IV)	96 (15.6%)

LVSI, lymphovascular space invasion. ‘Unknown’ denotes missing data in medical records.

**Figure 1:**
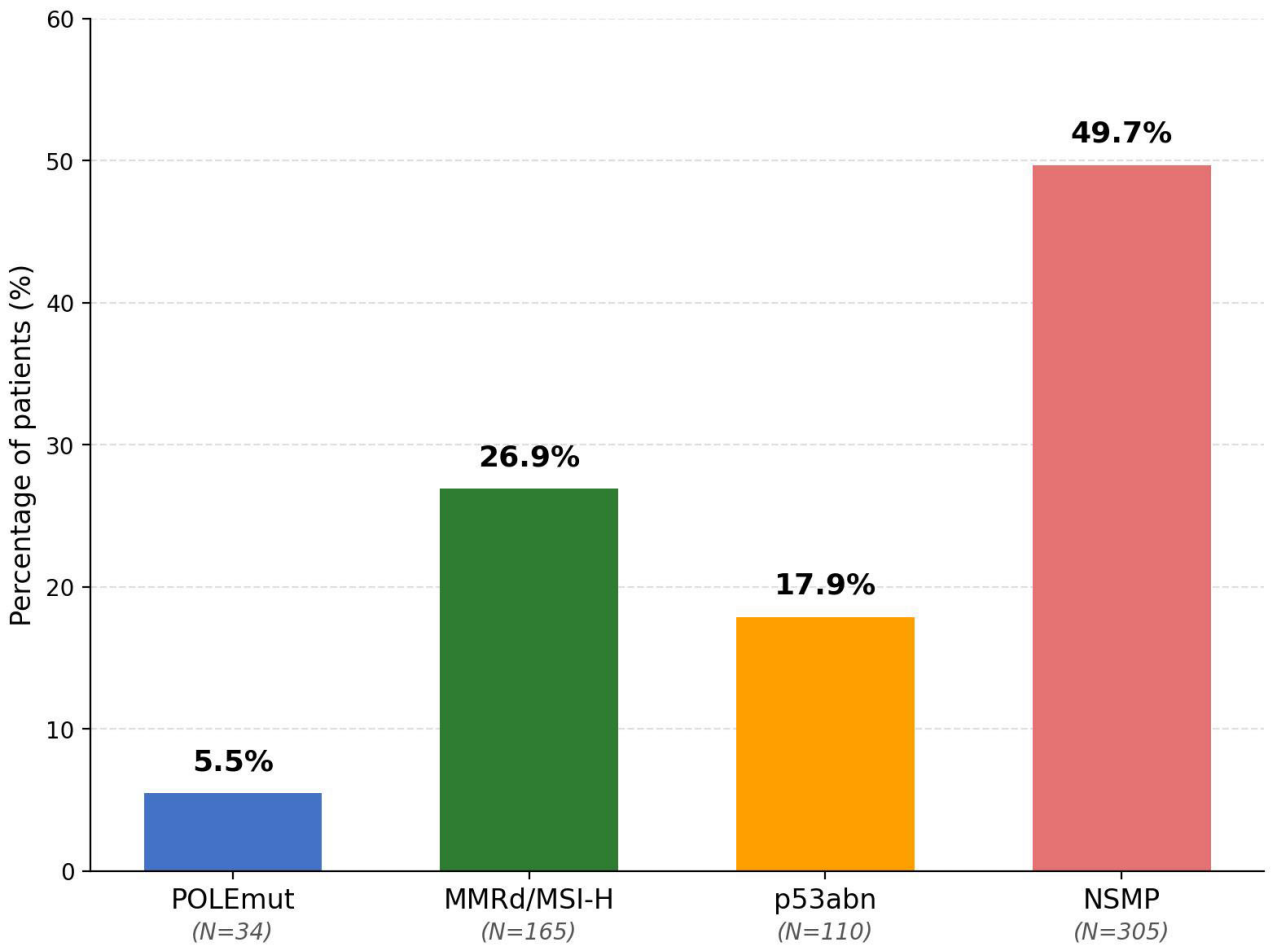
Distribution of molecular subtypes of endometrial cancer in the two-centre cohort (N = 614). MSI-H, high microsatellite instability; p53abn, p53 abnormal; NSMP, no specific molecular profile; MMRd, mismatch repair deficient; POLEmut, POLE-mutated.

The most frequent molecular subtype was NSMP (N = 305, 49.7%), followed by MMRd/MSI-H (N = 165, 26.9%), p53abn (N = 110, 17.9%), and POLEmut (N = 34, 5.5%) (**Figure 1**).

In stage-based analysis, p53abn was the only subtype significantly associated with advanced disease (OR 1.79; 95% CI 1.06–3.03; p=0.031). No statistically significant associations were observed for the remaining subtypes: POLEmut showed a non-significant trend towards advanced stage (OR 2.04; 95% CI 0.92–4.52; p=0.073), MMRd/MSI-H towards early stage (OR 0.63; 95% CI 0.37–1.08; p=0.088), and NSMP showed no association (OR 0.79; 95% CI 0.51–1.22; p=0.363). In the four-stage FIGO analysis, the proportion of p53abn increased progressively from 13.8% in stage I to 36.4% in stage IV (p<0.001). Detailed subtype distribution by stage is presented in **Table 2**.

**Table 2:** Distribution of molecular subtypes by FIGO stage (N = 614).

Part A: detailed distribution by FIGO stage					
Molecular subtype	FIGO I (N=369)	FIGO II (N=149)	FIGO III (N=85)	FIGO IV (N=11)	p value
POLEmut	23 (6.2%)	2 (1.3%)	8 (9.4%)	1 (9.1%)	0.008
p53abn	51 (13.8%)	34 (22.8%)	21 (24.7%)	4 (36.4%)	<0.001
MMRd/MSI-H	106 (28.7%)	40 (26.8%)	17 (20.0%)	2 (18.2%)	0.112
NSMP	189 (51.2%)	73 (49.0%)	39 (45.9%)	4 (36.4%)	0.891

Part B: two-level analysis — early vs. advanced stage					
Molecular subtype	Early I–II (N=518)	Advanced III–IV (N=96)		OR (95% CI)	p value
POLEmut	25 (4.8%)	9 (9.4%)		2.04 (0.92–4.52)	0.073
p53abn	85 (16.4%)	25 (26.0%)		1.79 (1.06–3.03)	0.031*
MMRd/MSI-H	146 (28.2%)	19 (19.8%)		0.63 (0.37–1.08)	0.088
NSMP	262 (50.6%)	43 (44.8%)		0.79 (0.51–1.22)	0.289

Part A: chi-squared test or Fisher's exact test as appropriate. Part B: univariable binary logistic regression; early stage (FIGO I–II) as reference group. OR, odds ratio; CI, confidence interval; *statistically significant (p<0.05). MSI-H, high microsatellite instability; p53abn, p53 abnormal; NSMP, no specific molecular profile; MMRd, mismatch repair deficient.

Among the 110 patients with p53 abnormalities identified by p53 IHC and/or *TP53* NGS, 23 discordant cases (20.9%) were identified between IHC assessment and *TP53* NGS analysis (for the whole group of 614 patients, discordant cases accounted for 3.7%). These were classified into seven mechanistic groups (**Table 3**). Clinicopathological characteristics of the 110 patients with p53 abnormalities and of the 23 discordant cases are presented in **Tables 4** and **5**, respectively. Case-level data for all discordant cases including tissue block, formalin fixation time, antibody clone, and VAF are provided in **Supplementary Table 1**. Among the 23 discordant cases, the final hierarchical ProMisE classification assigned 13 tumours (56.5%) to the NSMP subtype, 8 (34.8%) to the MMRd/MSI-H subtype, and 2 (8.7%) to the POLEmut subtype (**Table 6**). No discordant case remained classified as p53abn after application of the hierarchical ProMisE algorithm. Discordances occurring within MMRd/MSI-H and POLEmut tumours represented secondary *TP53* alterations that did not determine final molecular classification.

**Table 3:** Patterns of discordance between p53 IHC and *TP53* NGS results and their potential clinical interpretation (N = 23).

Gr.	N	IHC pattern	NGS result	Possible mechanism	Potential clinical implication
1	3	Wild type (~10% nuclear expression)	Nonsense/frameshift mutation (VAF 23–35%)	Masking of null phenotype by wild-type cells despite high mutant cell fraction	Risk of discordant wild-type IHC phenotype despite underlying *TP53* mutation, even at substantial VAF
2	4	Very low expression (<1–2%)	Truncating mutation (nonsense/frameshift/splicing)	Borderline interpretation between null phenotype and wild type	Potential discordance between IHC-based and mutation-based classification in borderline null phenotype cases
3a	4	Subpopulation (~5%) with p53 overexpression	Variable (wt/missense/nonsense/splicing)	Subclonal *TP53* alteration below NGS detection threshold	Uncertain biological significance; interpret within molecular context
3b	2	Subpopulation (~20%) with p53 overexpression	Wild type	p53 stabilisation without *TP53* mutation or spatial tumour heterogeneity	Potential discordance between IHC phenotype and *TP53* mutation status due to spatial heterogeneity or non-mutational p53 stabilisation
4	2	Wild type pattern (60–70% expression)	Missense mutation (low VAF 13–14%)	Subclonal *TP53* mutation with limited effect on protein stability	Potential discordance between genotype and IHC phenotype
5	1	Diffuse overexpression (100%)	Wild type	Non-mutational p53 stabilisation (e.g. MDM2 pathway dysregulation)	Potential discordance between IHC phenotype and *TP53* mutation status due to non-mutational p53 stabilisation
6	5	Borderline expression (~80%)	Wild type or missense mutation	Threshold interpretation around overexpression cut-off	Borderline IHC phenotype with potential discordance between IHC- and mutation-based classification, requiring careful pathological review
7	2	Wild type pattern (30–70% expression)	Nonsense mutation	Masking of null phenotype by wild-type tumour cells	Risk of discordant wild-type IHC phenotype despite underlying *TP53* alteration
Total	23				

Wt, wild type; IHC, immunohistochemistry; NGS, next-generation sequencing; VAF, variant allele frequency. A tumour subpopulation is defined as a spatially distinct area showing an aberrant p53 staining pattern comprising ≥5% of tumour cells. Total discordant cases: N = 23 (20.9% of 110 patients with p53 abnormalities identified by p53 IHC and/or TP53 NGS in whom both IHC and NGS were performed).

**Table 4:** Clinicopathological characteristics of patients with a p53 abnormality by p53 IHC and/or *TP53* NGS (N = 110).

Parameter	Category	N	% of N
Age at surgery	Mean ± SD	67.8 ± 10.2	—
	< 60 years	23	21.3%
	60–70 years	32	29.6%
	> 70 years	53	49.1%
>Histology	Endometrioid	78	70.9%
	Non-endometrioid	32	29.1%
>LVSI	None/focal	62	58.5%
	Substantial	44	41.5%
>FIGO stage	I	51	46.4%
	II	34	30.9%
	III	21	19.1%
	IV	4	3.6%
	Early (I–II)	85	77.3%
	Advanced (III–IV)	25	22.7%

Age calculated from PESEL number at time of surgery; available for 108/110 patients. LVSI available for 106/110 patients. FIGO stage available for all 110 patients; consistent with the p53abn row of **Table 2**. LVSI, lymphovascular space invasion.

**Table 5:** Clinicopathological characteristics of discordant cases (N = 23).

Parameter	Category	N	% of 23
Age at surgery	Mean ± SD	70.5 ± 9.8	—
	< 60 years	3	13.0%
	60–70 years	6	26.1%
	> 70 years	13	56.5%
Histology	Endometrioid	18	78.3%
	Non-endometrioid	5	21.7%
LVSI	None/focal	13	59.1%
	Substantial	9	40.9%
FIGO stage	I	9	39.1%
	II	6	26.1%
	III	7	30.4%
	IV	1	4.3%
	Early (I–II)	15	65.2%
	Advanced (III–IV)	8	34.8%

Age available for 22/23 patients. LVSI available for 22/23 patients; percentages calculated on available data. LVSI, lymphovascular space invasion.

**Table 6:** Distribution of discordant cases (N = 23) by discordance group and final hierarchical ProMisE molecular subtype.

Discordance group	POLEmut	MMRd/MSI-H	NSMP	Total
Group 1 (n=3)	0	2	1	3
Group 2 (n=4)	0	1	3	4
Group 3a (n=4)	1	1	2	4
Group 3b (n=2)	0	0	2	2
Group 4 (n=2)	0	0	2	2
Group 5 (n=1)	0	0	1	1
Group 6 (n=5)	0	3	2	5
Group 7 (n=2)	1	1	0	2
Total (N = 23)	2	8	13	23

ProMisE subtype was assigned according to the hierarchical algorithm (POLEmut > MMRd/MSI-H > p53abn). All 23 cases originated from the p53-abnormal subgroup (defined by IHC and/or NGS). No discordant case belonged to the p53abn-only category after application of the hierarchical ProMisE algorithm; all cases were ultimately assigned to POLEmut, MMRd/MSI-H, or NSMP according to the presence or absence of higher-hierarchy molecular alterations. MMRd, mismatch repair deficient; MSI-H, high microsatellite instability; NSMP, no specific molecular profile; POLEmut, POLE-mutated.

The most frequent source of discordance was difficulty in interpreting p53 IHC expression thresholds, involving both the lower 1% boundary (Groups 1, 2 and 7; 9 cases) and the upper 80% boundary (Group 6; 5 cases), together accounting for 60.9% of discordant cases. Other mechanisms included intratumoral heterogeneity with subclonal p53 expression (Groups 3a/3b; n=6), low-VAF *TP53* mutations (Group 4; n=2), and *TP53*-independent p53 stabilisation (Group 5; n=1).

**Figure 2:**
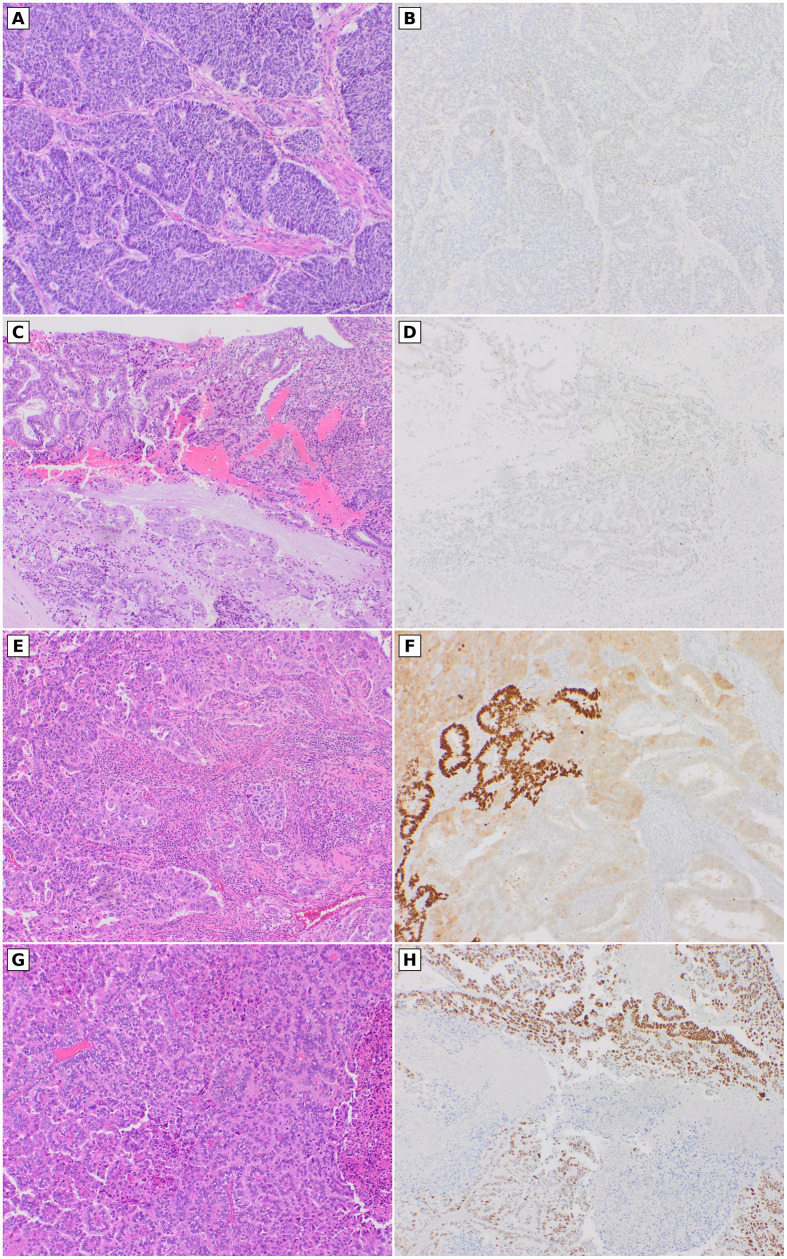
Representative histological and immunohistochemical findings in discordance Groups 1–3b. Corresponding haematoxylin–eosin (HE) staining and p53 immunohistochemistry (IHC) are shown for each group. **(A, B)** Group 1 (Case 1): wild-type p53 IHC pattern (~10% nuclear expression) in the presence of a nonsense *TP53* mutation (VAF 35%) by NGS, consistent with masking of null phenotype by wild-type tumour cells. **(C, D)** Group 2 (Case 15): very low p53 expression (~2% of tumour cells), categorised as wild type by IHC despite a truncating *TP53* mutation on NGS; borderline interpretation at the null-phenotype threshold. **(E, F)** Group 3a (Case 17): spatially distinct subpopulation (~5% of tumour cells) with aberrant p53 overexpression, with a splicing *TP53* mutation (VAF 48%) detected by NGS; consistent with subclonal *TP53* alteration. **(G, H)** Group 3b (Case 10): subpopulation (~20% of tumour cells) with p53 overexpression in the absence of a detectable *TP53* mutation by NGS; consistent with non-mutational p53 stabilisation or spatial tumour heterogeneity. HE, haematoxylin–eosin; IHC, immunohistochemistry; NGS, next-generation sequencing; VAF, variant allele frequency. Original magnification ×200.

**Figure 3:**
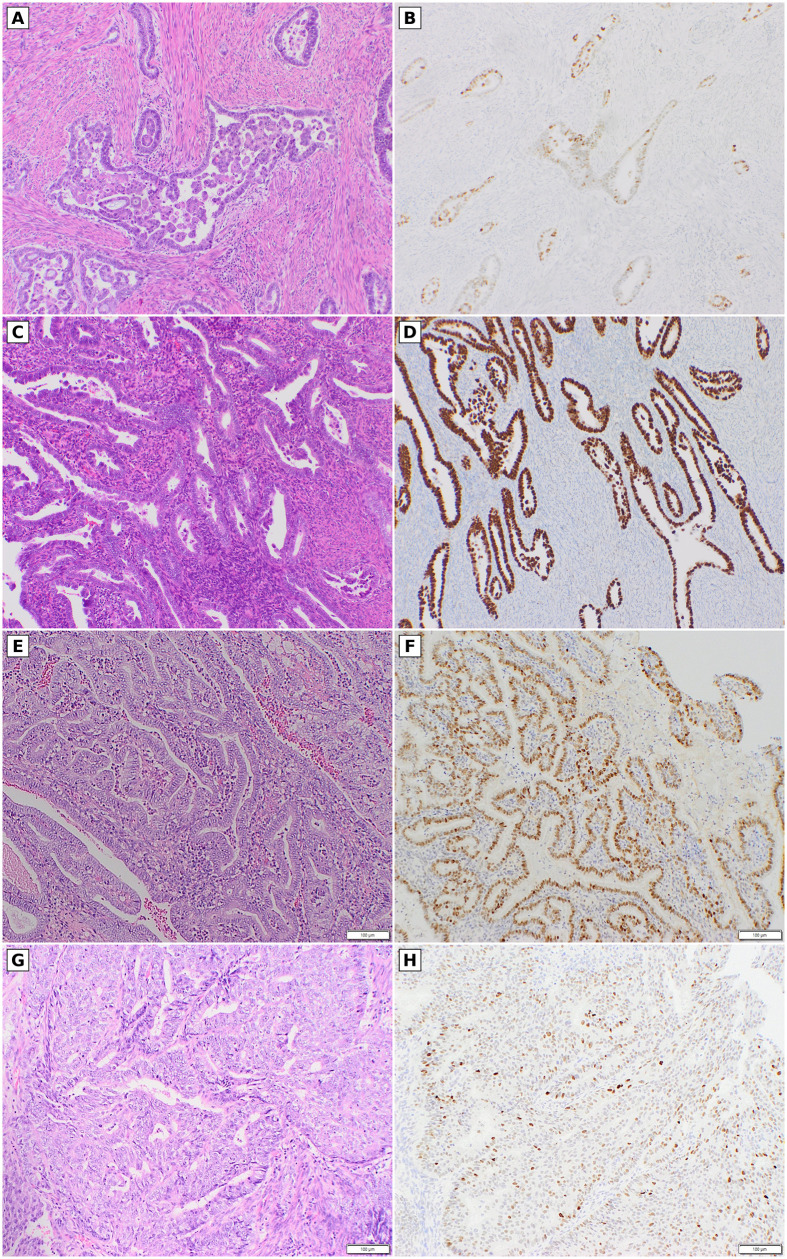
Representative histological and immunohistochemical findings in discordance Groups 4–7. Corresponding haematoxylin–eosin (HE) staining and p53 immunohistochemistry (IHC) are shown for each group. **(A, B)** Group 4 (Case 6): wild-type p53 IHC pattern (60% nuclear expression) with a missense *TP53* mutation at low variant allele frequency (VAF 13%) by NGS; consistent with a subclonal mutation with limited effect on protein stability. **(C, D)** Group 5 (Case 13): diffuse p53 overexpression (100% of tumour cells) in the absence of a detectable *TP53* mutation by NGS; consistent with non-mutational p53 stabilisation, potentially via MDM2 pathway dysregulation. **(E, F)** Group 6 (Case 21): borderline p53 expression (~80% of tumour cells) with wild-type *TP53* by NGS; illustrating the interpretive challenge at the overexpression threshold. **(G, H)** Group 7 (Case 23): wild-type p53 IHC pattern (65% nuclear expression) with a nonsense *TP53* mutation (VAF 9%) by NGS; consistent with masking of null phenotype by wild-type tumour cells at low mutant cell fraction. HE, haematoxylin–eosin; IHC, immunohistochemistry; NGS, next-generation sequencing; VAF, variant allele frequency. Original magnification ×200.

In Group 1, the IHC pattern was interpreted as wild type despite nonsense or frameshift mutations detected by NGS with a VAF of 23–35%, indicating masking of the null phenotype by surrounding wild-type cells (**Figures 2A, B**). Group 2 comprised four cases with very low p53 expression (<1–2%), all carrying truncating *TP53* mutations confirmed by NGS (**Figures 2C, D**). Six cases in Group 3 demonstrated tumour cell subpopulations with p53 overexpression (≥5% of cells); NGS results were heterogeneous (**Figure 2E–H**). In Group 4 (n=2), IHC showed a wild-type pattern (60–70%) whilst NGS detected a missense mutation with low VAF (13–14%) (**Figures 3A, B**). Group 5 comprised a single case with diffuse overexpression (100%) without a detectable *TP53* mutation (**Figures 3C, D**). Group 6 included five cases with p53 expression around the 80% threshold; NGS was wild-type in three cases and revealed a missense mutation in two (**Figures 3E, F**). Group 7 showed a wild-type IHC pattern (30–70%) with nonsense mutations detected by NGS (**Figures 3G, H**). No patient received preoperative radiotherapy or chemotherapy; histological heterogeneity was present in four discordant cases.

## Discussion

### Summary of main results

In this two-centre retrospective study of 614 patients with endometrial cancer — all of whom underwent both p53 IHC and *TP53* NGS — IHC–NGS discordance was identified in 20.9% of cases within the subgroup with p53 abnormalities identified by p53 IHC and/or *TP53* NGS (23/110). Seven mechanistic patterns of discordance were distinguished, with threshold interpretation difficulties accounting for the majority (60.9%). The p53abn subtype was the only molecular subtype significantly associated with advanced-stage disease (OR 1.79; 95% CI 1.06–3.03; p=0.031), with its frequency increasing progressively from 13.8% in stage I to 36.4% in stage IV. These findings are consistent with published data confirming the association of p53abn with advanced stage and distant recurrence (9, 10, 32), including in early-stage and non-myoinvasive disease where p53abn retains independent prognostic value (11). These data confirm that accurate p53 classification carries direct therapeutic consequences in endometrial cancer. The principal contribution of this study lies not in the observation of discordance itself, but in its systematic organisation into clinically interpretable mechanistic categories.

### Results in the context of published literature

The relatively high discordance rate of 20.9% reflects the deliberate restriction of the analysis to the p53-abnormal subgroup rather than the overall frequency of IHC–NGS discordance in an unselected endometrial cancer population; it should not be interpreted as indicating that one in five endometrial cancers will yield discordant p53 results. Nonetheless, the observed rate is consistent with or slightly higher than values reported in the literature for p53-assessed cases (4, 5), most likely reflecting underlying tumour heterogeneity and subclonal evolution rather than methodological limitations alone (4, 16). It should be emphasised that in this study, all discordant cases underwent re-evaluation by a pathologist experienced in gynaecological pathology; the case-level data presented in **Supplementary Table 1** confirm that biological sources of discordance (heterogeneous VAF profiles, subclonal IHC patterns) are identifiable independently of staining platform or antibody clone. Accordingly, while the absence of formal centralised IHC review is acknowledged as a limitation, the structured re-review performed for this study provides a degree of internal validation for the mechanistic interpretations proposed.

p53 immunohistochemistry reflects functional protein status and remains the cornerstone of molecular classification, whilst NGS — although often considered a reference method for *TP53* mutation detection — assesses a different biological dimension (4, 18). Within the original ProMisE framework, p53abn classification is based on immunohistochemical phenotype, although newer versions (ProMisE NGS) recognise *TP53* mutation as an equivalent criterion, demonstrating high concordance with the IHC-based approach (κ=0.96) (18, 19). Cases with discordant IHC–NGS results represent a diagnostically heterogeneous group whose clinical and biological significance is increasingly recognised in the literature (20–22). The predominance of NSMP tumours amongst discordant cases (13/23, 56.5%) suggests that IHC–NGS discrepancies may be of greatest clinical importance precisely in the subgroup in which p53 status serves as the principal determinant of molecular classification and treatment allocation; discordances within MMRd/MSI-H and POLEmut tumours, by contrast, do not alter final classification under the ProMisE hierarchy and are therefore less likely to affect therapeutic decisions.

The most common source of discordance — difficulties in interpreting IHC threshold values — has been well documented in the literature. The limited reproducibility of semi-quantitative assessment near the 1% and 80% boundaries arises from the inherently subjective nature of such evaluation (3, 4). Data from the PORTEC-3 cohort confirm that IHC–NGS concordance increases after exclusion of POLEmut and MMRd tumours (from 93% to 96%), highlighting the importance of molecular context (6).

The masking of null phenotype by wild-type cells is particularly striking in Group 1, where VAF values of 23–35% indicate that a substantial mutant cell fraction was present yet remained undetected by IHC. This phenomenon has been described in endometrial cancer by Singh et al. (4), and analogous mechanisms have been documented in breast and colorectal cancer, where admixture of wild-type stromal or epithelial cells can suppress the IHC signal from a mutant clone. The VAF-to-cell-fraction relationship depends on zygosity: under heterozygosity, a VAF of 23–35% corresponds to an estimated 46–70% mutant cell fraction, supporting the interpretation that masking rather than low clonal burden accounts for the false-negative IHC result in these cases.

The identification of subclonal p53 expression in Group 3 is consistent with previous observations that in POLEmut and MMRd tumours, subclonal *TP53* alterations are relatively frequent secondary events (6, 15, 16). According to the ProMisE hierarchy, such alterations should not lead to classification as p53abn. Multicentre data indicate that coexisting *TP53* abnormalities in these subtypes may be associated with an increased frequency of lymph node metastases (14, 17), which may suggest a model of clonal evolution in which subclonal *TP53* alterations are acquired during tumour progression — although the available evidence is insufficient to confirm this mechanism and prospective validation is required. The interpretation of limited areas of increased p53 staining, as in Group 3b, warrants particular caution. In our Group 3b cases, the subpopulations with increased staining were not accompanied by a detectable *TP53* mutation on NGS. Such limited foci are inherently susceptible to interobserver variation, and small areas interpreted as subclonal overexpression may in some cases reflect differences in interpretation rather than genuine biological subclonality. A recent study proposed that subclonal mutant-pattern p53 staining involving ≥10% of tumour cells may support p53abn classification in otherwise-NSMP tumours and may be associated with less favourable outcomes (16). The clinical and prognostic significance of these limited abnormal areas is not yet established and is not currently incorporated into the ProMisE framework. Group 5 — diffuse p53 overexpression without a detectable *TP53* mutation — illustrates alternative protein stabilisation mechanisms, including dysregulation of the MDM2–p53 axis (3, 4, 33).

### Strengths and weaknesses

The principal strength of this study is the systematic, mechanistic classification of IHC–NGS discordances into seven groups, providing a structured interpretative framework that may facilitate the management of diagnostically ambiguous cases in routine practice. The two-centre design and the case-level re-review by a gynaecological pathologist partially limit inter-laboratory variability. The main limitations include the retrospective design, the absence of formalised centralised IHC review, and the use of two different antibody clones and staining platforms across centres. Pre-analytical conditions, including fixation duration, were not formally standardised between the two centres and reflected routine institutional practice at each site.

The discordance analysis was restricted to the subgroup with p53 abnormalities identified by p53 IHC and/or *TP53* NGS (n=110), as this is the clinically relevant context for IHC–NGS discordance; the representativeness of this subgroup for the broader population of endometrial cancer patients is inherently constrained by the molecular subtype distribution of the cohort. The absence of long-term follow-up and survival data precludes assessment of the prognostic impact of the identified discordance patterns.

### Implications for practice and future research

Cases with p53 expression near the 1% and 80% thresholds should be flagged as diagnostically uncertain and referred for review by a second pathologist with expertise in gynaecological pathology. In clinically significant scenarios, supplementary NGS analysis of *TP53* should be considered before therapeutic decisions are finalised. This framework is intended as a diagnostic support tool rather than a redefinition of molecular classification. The molecular profile of endometrial cancer is generally stable between primary diagnosis and recurrence, with ProMisE subtype discordance affecting only a minority of recurrent cases (13, 24, 25); nevertheless, cases with borderline p53 classification at primary diagnosis may represent a subset at particular risk of apparent subtype reclassification, underscoring the importance of accurate initial assessment. Prospective data from PORTEC-4a confirm that an integrated molecular profile enables individualisation of adjuvant treatment (27). Ongoing prospective trials — RAINBO p53-Red (NCT05255653) and PETREC (NCT05655260) — are directly dependent on precise p53 classification (28, 29). Future studies with long-term survival follow-up are needed to establish whether the discordance patterns identified here carry independent prognostic significance.

## Conclusion

IHC–NGS discordances in p53 assessment constitute a multimechanistic phenomenon observed in over 20% of p53-abnormal cases, with difficulties in threshold interpretation accounting for the majority of discordances. The proposed seven-group mechanistic framework provides a structured approach for interpreting discordant results in routine diagnostic practice. IHC-based molecular classification remains the foundation of treatment decisions in endometrial cancer; however, its reliable application requires awareness of the biological mechanisms underlying discordance, integration with full molecular context, and a low threshold for pathological re-evaluation in borderline cases.

## Data Availability

The datasets presented in this study can be found in online repositories. The names of the repository/repositories and accession number(s) can be found in the article/**Supplementary Material**.

## Supplementary material

Supplementary Table 1 Case-level characteristics of discordant p53 IHC–*TP53* NGS results (N = 23).
