# Supplementary material for: Interpreting p53 IHC–TP53 NGS discordance in endometrial cancer: a mechanism-based diagnostic framework and clinical implications
Source: Front Oncol. 2026 Jul 22;16:1862855. doi: 10.3389/fonc.2026.1862855 (PMC13437294; doi:10.3389/fonc.2026.1862855)
Supplement: Supplementary file 1 [file SupplementaryFile1.pdf]

Supplementary Table S1. Case-level characteristics of discordant p53 IHC–*TP53* NGS results (N=23)

Case	Formalin fixation	Tissue block (IHC/NGS)	Histol. heterog.	p53 clone	IHC: % stained cells	TP53 mutation	VAF (%)	Mutation type
Group 1 (n=3) — IHC: wild type (~10%); NGS: nonsense/frameshift (VAF 23–35%). Mechanism: masking of null phenotype by wild-type cells.								
1	4d 6h	Same block	Absent	BP53-11	10%	Present	35	Nonsene
2	3d 5h	Different block, same specimen	Absent	BP53-11	10%	Present	27	Nonsense
16	5d 22h	Same block	Absent	BP53-11	10%	Present	23	Frameshift
Group 2 (n=4) — IHC: very low expression (<1–2%); NGS: truncating mutation. Mechanism: borderline null phenotype interpretation.								
3	2d 7h	Same block	Absent	BP53-11	1%	Present	9; 63.4	Nonsense; splicing
7	2d 5h	Different block, same specimen	Absent	BP53-11	1%	Present	37	Nonsense
9	1d 6h	Same block	Absent	BP53-11	<1%	Present	53	Frameshift
15	1d 21h	Different block, same specimen	Absent	BP53-11	2%	Present	10	Nonsense
Group 3a (n=4) — IHC: subpopulation (~5%) overexpression; NGS: variable. Mechanism: subclonal TP53 alteration below NGS threshold.								
4	2d 3h	Same block	Absent	BP53-11	5% (overexpr.)	Present	71	Missense

Case	Formalin fixation	Tissue block (IHC/NGS)	Histol. heterog.	p53 clone	IHC: % stained cells	TP53 mutation	VAF (%)	Mutation type
5	3d 6h	Same block	Absent	BP53-11	5% (overexpr.)	Absent	—	—
8	2d 4h	Different block, same specimen	Absent	BP53-11	5% (overexpr.)	Present	5	Nonsense
17	2d 1h	Same block	Absent	BP53-11	5% (overexpr.)	Present	48	Splicing
Group 3b (n=2) — IHC: subpopulation (~20%) overexpression; NGS: wild type. Mechanism: p53 stabilisation without TP53 mutation or spatial heterogeneity.								
10	3d 3h	Same block	Present	BP53-11	20% (overexpr.)	Absent	—	—
19	1d	Same block	Absent	DO-7	20% (overexpr.)	Absent	—	—
Group 4 (n=2) — IHC: wild-type (60–70%); NGS: missense (low VAF 13–14%). Mechanism: subclonal TP53 mutation with limited effect on protein stability.								
6	3d 3h	Same block	Absent	BP53-11	60%	Present	13	Missense
18	2d 23h	Different block, same specimen	Absent	BP53-11	70%	Present	14	Missense
Group 5 (n=1) — IHC: diffuse overexpression (100%); NGS: wild type. Mechanism: non-mutational p53 stabilisation (e.g. MDM2 dysregulation).								
13	6d 22h	Different block, same specimen	Present	BP53-11	100%	Absent	—	—
Group 6 (n=5) — IHC: borderline expression (~80%); NGS: wild type or missense. Mechanism: threshold interpretation around overexpression cut-off.								

Case	Formalin fixation	Tissue block (IHC/NGS)	Histol. heterog.	p53 clone	IHC: % stained cells	TP53 mutation	VAF (%)	Mutation type
11	3d 1h	Different block, same specimen	Absent	BP53-11	80%	Present	6	Missense
12	6d 23h	Different block, same specimen	Absent	BP53-11	80%	Absent	—	—
14	6d 22h	Different block, same specimen	Absent	BP53-11	80%	Present	9	Missense
20	1d	Diff. specimens (IHC resection, NGS biopsy)	Absent	DO-7	>80%	Absent	—	—
21	23h	Same block	Absent	DO-7	>80%	Absent	—	—
Group 7 (n=2) — IHC: wild-type (30–70%); NGS: nonsense mutation. Mechanism: masking of null phenotype by wild-type tumour cells.								
22	1d 1h	Different block, same specimen	Present	DO-7	35%	Present	29	Nonsense
23	2d	Same block	Present	DO-7	65%	Present	9	Nonsense

All discordant cases (N=23) with case-level IHC and NGS data. Tissue block indicates whether p53 IHC and TP53 NGS were performed on the same block, on different blocks from the same surgical specimen, or on different specimens (one case: IHC on resection, NGS on biopsy). p53 clone: BP53-11 (Centre 1, n=18) or DO-7 (Centre 2, n=5). IHC values denote the percentage of tumour cells with nuclear staining; for subclonal patterns, the size of the aberrant overexpressing subpopulation is given. VAF — variant allele frequency. —, not applicable (no mutation detected). Formalin fixation time

ranged from 23 h to 6 days 23 h. No patient received preoperative radiotherapy or chemotherapy. Histological heterogeneity was present in four cases (cases 10, 13, 22, 23).
